# Challenges and Opportunities for Grounding Cognition

**DOI:** 10.5334/joc.116

**Published:** 2020-09-29

**Authors:** Lawrence W. Barsalou

**Affiliations:** 1 Institute of Neuroscience and Psychology, School of Psychology, University of Glasgow, Glasgow, UK

**Keywords:** Action, Categorisation, Embodied cognition, Emotion and cognition, Event cognition, Semantics

## Abstract

According to the grounded perspective, cognition emerges from the interaction of classic cognitive processes with the modalities, the body, and the environment. Rather than being an autonomous impenetrable module, cognition incorporates these other domains intrinsically into its operation. The Situated Action Cycle offers one way of understanding how the modalities, the body, and the environment become integrated to ground cognition. Seven challenges and opportunities are raised for this perspective: (1) How does cognition emerge from the Situated Action Cycle and in turn support it? (2) How can we move beyond simply equating embodiment with action, additionally establishing how embodiment arises in the autonomic, neuroendocrine, immune, cardiovascular, respiratory, digestive, and integumentary systems? (3) How can we better understand the mechanisms underlying multimodal simulation, its functions across the Situated Action Cycle, and its integration with other representational systems? (4) How can we develop and assess theoretical accounts of symbolic processing from the grounded perspective (perhaps using the construct of simulators)? (5) How can we move beyond the simplistic distinction between concrete and abstract concepts, instead addressing how concepts about the external and internal worlds pattern to support the Situated Action Cycle? (6) How do individual differences emerge from different populations of situational memories as the Situated Action Cycle manifests itself differently across individuals? (7) How can constructs from grounded cognition provide insight into the replication and generalization crises, perhaps from a quantum perspective on mechanisms (as exemplified by simulators).

## 1. Domains underlying grounded cognition

Cognition has traditionally been viewed as a module in the brain (Fodor, 1975; Pylyshyn, 1984). From this perspective, a module for cognition operates separately and independently of other modules for vision, audition, action, emotion, and so forth. Although modules pass information between themselves, each operates autonomously, with its internal computations unaffected by activity in the others. Some call this approach ‘the sandwich model’ (Hurley, 2001), with cognition sandwiched between perception and action. Whereas perception primarily serves to provide input into the cognitive module, action primarily serves to get information out. Otherwise, perception and action play no critical roles in the computations that constitute cognition. All cognitive processes are contained within the cognitive module, with other modules having no impact on these processes, other than the exchange of information. The fact that textbooks on cognition often only cover perception and action minimally, if at all, further attests to this state of affairs. Perhaps a more serious concern is that many cognitive psychologists, cognitive scientists, and neuroscientists at least implicitly endorse the modular approach (even when they might not explicitly hold it), focusing their research on cognitive processes without seriously taking perception and action into account (along with other phases of the Situated Action Cycle described later).

Much research challenges the modular approach. Perhaps most basically, the classic grounding problem raises the issue of how abstract amodal symbols—typically assumed intrinsic to the cognitive module—become mapped into perception and the world (Harnad, 1990; Searle, 1980). Much additional research suggests that cognition utilizes the perceptual modalities and the motor system for representation and processing purposes (for reviews and relevant collections, see Barsalou, 1999, 2008a, 2016b; Coello & Fischer, 2016a, 2016b; De Vega, Glenberg, & Graesser, 2008; Kemmerer, 2015; Kiefer & Barsalou, 2013; Kiefer & Pulvermüller, 2012; Martin, 2007, 2016; Meteyard, Cuadrado, Bahrami, & Vigliocco, 2012; Pecher & Zwaan, 2005; Pulvermüller, 1999, 2005, 2013). Still other work proposes that cognition emerges from coupling of the brain, body, and environment (e.g., Aydede & Robbins, 2009; Barsalou, 2019; Barsalou, Dutriaux, & Scheepers, 2018; Dutriaux, Clark, Papies, Scheepers, & Barsalou, 2019; Gibson, 1966, 1979; Hutchins, 1995; Newen, Bruin, & Gallagher, 2018; Thompson, 2010; Varela, Thompson, & Rosch, 2016). Finally, considerable empirical work demonstrates that higher-level cognitive processes penetrate deeply into the activity of perceptual systems, affecting their computations (Clark, 2013; Marslen-Wilson & Tyler, 1980; McClelland & Rumelhart, 1981; Muckli et al., 2015; Muckli & Petro, 2017; Murray, Boyaci, & Kersten, 2006; Rumelhart & McClelland, 1982; Samuel, 1997; Smith & Muckli, 2010). From the perspective of all this work, it appears increasingly difficult to defend the position that an autonomous impenetrable module in the brain implements cognition.

### 1.1. What to call it?

Often the non-modular perspective is referred to as *embodied cognition*. This description is problematic for two reasons. First, some researchers take ‘embodied’ to imply incorrectly that the body must necessarily be engaged during cognition (e.g., Markman & Brendl, 2005; but see Dantzig, Zeelenberg, & Pecher, 2009). Second, and more significantly, the body offers only *one* form of grounding. Other important forms of grounding exist as well, including the modalities, the physical environment, and the social environment.

**Figure 1:**
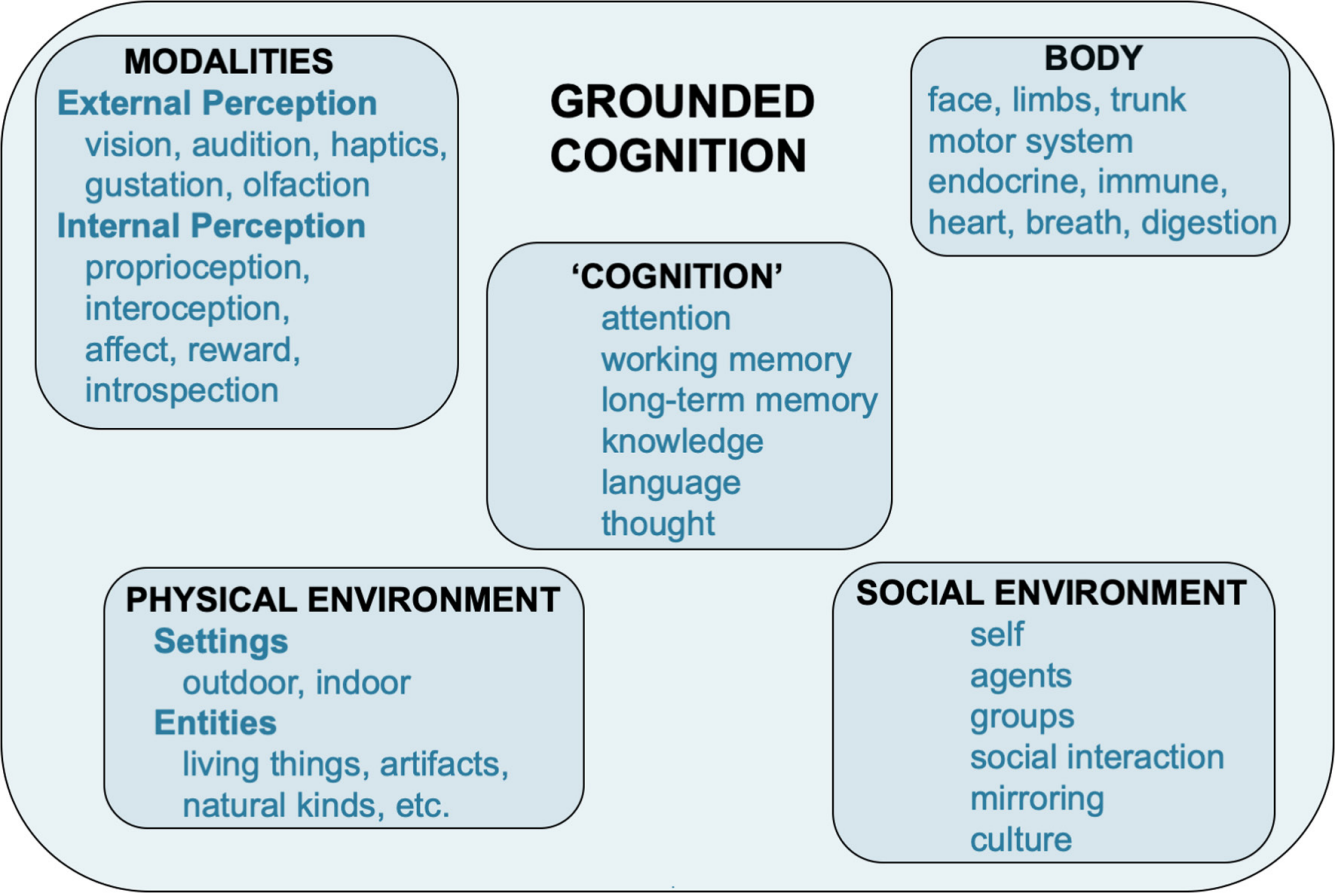
Domains of grounded cognition. Cognition emerges from grounding classic cognitive mechanisms in the perceptual modalities, body, physical environment, and social environment.

For these reasons, researchers often refer to the non-modular perspective more broadly, calling it *grounded cognition* (Barsalou, 2008a, 2010, 2016d; Kiefer & Barsalou, 2013; Pecher & Zwaan, 2005) and *4E cognition* (cognition that is *embodied, embedded, enactive, and extended*; Newen et al., 2018; Thompson, 2010). Figure 1 illustrates this broader perspective. In the center are the classic kinds of processes that researchers often associate with cognition. Surrounding these processes are domains that ground them, including the modalities, the body, the physical environment, and the social environment. From the grounded perspective, cognition does not simply result from an isolated set of processes. Instead, cognition emerges from interactions of these processes with these four domains. From the 4E perspective, cognition, affect, and behavior emerge from the *body* being *embedded* in environments that *extend* cognition, as agents *enact* situated action reflecting their current cognitive and affective states.

For the remainder of this article, I will continue using *grounded cognition* when referring to this general approach, assuming that it naturally incorporates the 4Es. I will only use *embodied cognition* when referring to cognition being specifically grounded in the body.

## 2. Integrating the domains of grounded cognition with the Situated Action Cycle

As we just saw, one central theme of the grounded approach is that cognition emerges from interactions between classic cognitive processes, the modalities, the body, the physical environment, and the social environment (Figure 1). Cognition is not a module in the brain that can be studied effectively in isolation but must be studied in the context of these other domains. Another central theme is that cognition supports situated action (e.g., Clark, 1998; Glenberg, 1997). Cognition is not an end in itself but typically guides effective action in the world. Rather than simply being the culmination of bottom-up processing streams from the perceptual modalities, cognition operates as a mediator between perception and action (Barsalou, 2016a).

**Figure 2:**
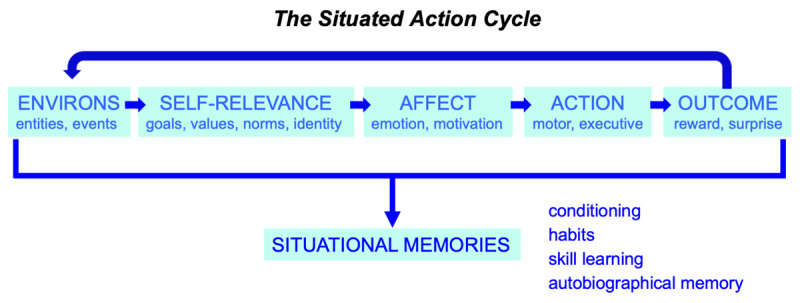
The Situated Action Cycle. An idealized series of discrete linear phases is shown, together with an iterative loop (top) and a situational memory process (bottom). In actual operation, phases may be smeared across each other in time and/or omitted. Also, various loops and alternative relations between phases may emerge. This idealized representation simply illustrates the critical phases and their approximate statistical relations.

The Situated Action Cycle offers one account of the relations between perception, cognition, action and other relevant domains, including the environment, affect, and outcomes. Figure 2 illustrates the Situated Action Cycle as an idealized series of discrete linear phases. In actual operation, these phases may be smeared across each other in time and/or omitted. Also, various loops and alternative relations between phases may emerge. This idealized representation simply illustrates the critical phases and their approximate relations.

As Figure 2 illustrates, perceived entities and events in the environment typically initiate the Situated Action Cycle (although it can be initiated internally as well). Once an entity or event is perceived, its self-relevance for the agent is assessed, including its relations to the agent’s goals, values, identity, and relevant social norms (we assume that perception constitutes part of apprehending and computing self-relevance). Other cognitive processing of the entity or event may ensue as well, but most basically, cognitive processing establishes its relevance and meaning for the agent. In turn, self-relevance induces affect, expressed significantly in the body (embodiments). Affect often takes the form of emotion, contributing physical feelings that complement cognitive assessments of self-relevance. Affect also takes the form of motivation, as the appraisals and feelings associated with self-relevance induce impetus to promote the agent’s goals, values, identity, and conformity to norms. If motivation is sufficiently strong, these actions are initiated, ranging from eye movements and overt bodily actions (embodiments) to executive cognitive actions. Finally, actions produce outcomes, both in the external world (e.g., reward, punishment) and inside the agent (e.g., prediction error).

As the loop at the top of the Situated Action Cycle in Figure 2 illustrates, outcomes change the external and internal environments, triggering further iterations of the cycle. Over the course of daily activities, the cycle iterates continually, as agents evaluate and respond to changing environmental conditions. As much literature shows, motivations and actions in the cycle not only produce outcomes, they also influence how the environment is perceived during the self-relevance phase (e.g., Laitin, Tymoski, Tenhundfeld, & Witt, 2019; Proffitt, 2006, 2013; Tenhundfeld & Witt, 2017; Witt, Tenhundfeld, & Tymoski, 2017).

Finally, as Figure 2 illustrates at the bottom, each run of the Situated Action Cycle superimposes information about its operation across memory systems in the brain. Elsewhere, my colleagues and I have referred to these situational memories as *situated conceptualizations* (e.g., Barsalou, 2003b, 2009, 2016c, 2016d, 2019; Barsalou, Niedenthal, Barbey, & Ruppert, 2003; Lebois, Wilson-Mendenhall, Simmons, Barrett, & Barsalou, 2018; Wilson-Mendenhall, Barrett, Simmons, & Barsalou, 2011). To the extent that the Situated Action Cycle runs in a similar manner across a repeated kind of situation (e.g., using a hammer, preparing coffee, cooking with a friend), a well-entrenched pattern for implementing the cycle becomes established in memory to facilitate its implementation in similar future situations. We assume that the accumulation of such patterns underlies conditioning, habit learning, skill acquisition, and autobiographical memory.

The Situated Action Cycle is hardly a novel idea in psychology, cognitive science, and neuroscience. For the past century, variants of it have played central roles across disciplines. Behavioral conditioning, for example, offers a classic example of the Situated Action Cycle in psychology (e.g., Bouton & Todd, 2014; Domjan, 2014). As a behavior achieves a desired outcome, it becomes associated with predictive environmental cues that signal the availability of reward. To the extent that this pattern occurs repeatedly, the reward conditions the behavior in response to the situation.

Classic theories of goal pursuit in cognitive science offer a different take on the Situated Action Cycle, inserting internal cognitive and affective states between the environment, action, and outcomes central to the Behaviorist approach. In cognitive theories, goal-directed behavior becomes organized around a cycle that integrates the environment, cognition, action, and outcomes, similar to the Situated Action Cycle. In seminal work Miller, Galanter, and Pribram (1960) developed a theory of goal-directed behavior that later inspired major theories of problem solving (e.g., Newell & Simon, 1972) and production systems (e.g., Anderson, 1983; Newell, 1973). Reinforcement learning offers another classic example of this approach in cognitive science that emphasizes the importance of reward (e.g., Daw & Frank, 2009; Sutton & Barto, 1998), also central in neuroscience (e.g., Botvinick, Niv, & Barto, 2009; Ito & Doya, 2011; O’Doherty, 2012)

Theories of narrative structure and text processing propose that conceptual structures similar to the Situated Action Cycle organize people’s knowledge of events during event processing, autobiographical memory, and language comprehension (e.g., Baerger & McAdams, 1999; Edson Escalas, 2004; Reese et al., 2011; Stein & Hernandez, 2007). As people perceive, remember, and discuss events, they represent sequences that integrate the environment, cognition (self), emotion, action, and outcomes. Theories of conceptual processing have similarly proposed that knowledge becomes organized in this manner (e.g., Barsalou, 2003b, 2016c, d; Barsalou, Dutriaux, & Scheepers, 2018). Because the Situated Action Cycle captures an organized set of processes central to many activities and processes, its central role across disciplines is no accident.

**Figure 3:**
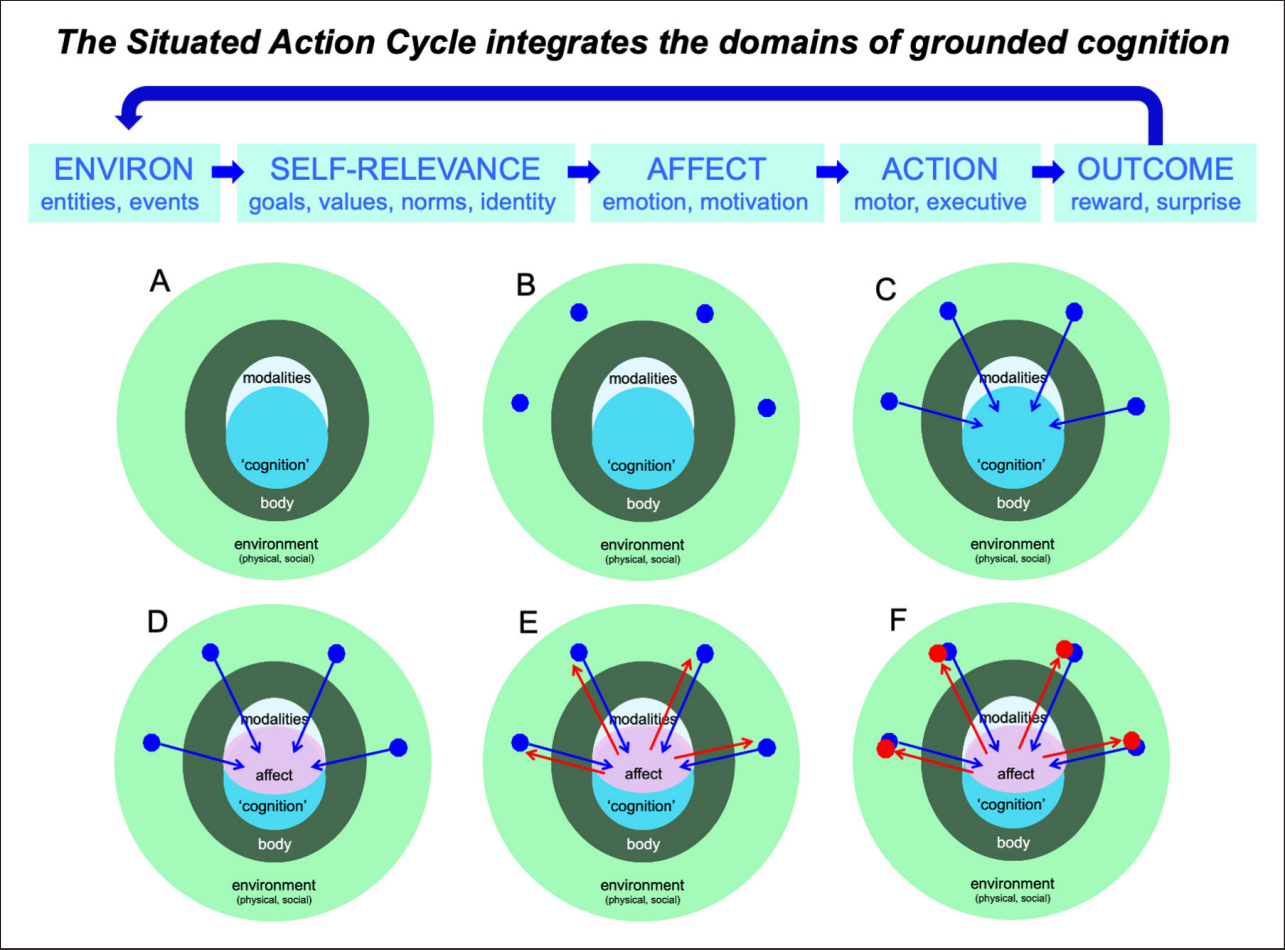
Illustration of how the Situated Action Cycle integrates the domains of grounded cognition. Panel A illustrates the five domains that underlie cognition in Figure 1: classic cognitive mechanisms (blue), the modalities (grey), the body (dark green), the physical and social environments (light green). Panel B illustrates the appearance of entities and events during the environmental phase of the cycle. Panel C illustrates perception of these entities and events via the modalities during the self-relevance phase, together with establishing their implications for the agent’s goals, values, identity, norms, etc. via cognitive processing. Panel D illustrates the resultant activation of emotion and motivation during the affect phase, often heavily expressed in the body. Panel E illustrates the production of behavior in the environment during the action phase, often resulting from emotion and motivation. Panel F illustrates the results of action during the outcome phase, including reward and assessments of prediction accuracy. As this figure illustrates overall, the Situated Action Cycle integrates the domains of grounded cognition, suggesting that these domains are important because, together, they implement the elements of situated action.

Interestingly, the Situated Action Cycle integrates the five domains that underlie grounded cognition in Figure 1. To see this integration, consider Figure 3. Panel A of Figure 3 illustrates the five domains that underlie grounded cognition, including classic cognitive processes (blue), the modalities (grey), the body (dark green), the physical and social environments (light green). Panel B illustrates the appearance of entities and events during the environmental phase of the cycle. Panel C illustrates the perception of these entities and events via the modalities during the self-relevance phase, together with establishing their implications for the agent’s goals, values, identity, and norms via cognitive processing. Panel D illustrates the resultant activation of emotion and motivation during the affect phase of the cycle, often expressed in the body. Panel E illustrates the production of behavior in the environment during the action phase, often resulting from emotion and motivation. Panel F illustrates the results of action during the outcome phase, including reward and assessment of prediction accuracy.

As Figure 3 illustrates, the Situated Action Cycle integrates the domains that underlie grounded cognition. Because, each domain helps implement the cycle—and indeed appears necessary for it—they all are important for understanding how cognition becomes grounded (Figures 1 and 2).

### 2.1. Related future issues

As cognitive psychologists, cognitive scientists, and neuroscientists study cognition, they often extract it from the Situated Action Cycle, ignoring the environment that couples with cognition, along with the affect, action, and outcomes that cognition produces. No doubt, it is convenient to isolate cognition in this manner such that it can be well controlled and elegantly modeled (Barsalou, 2019; Miller et al., 2019). Problematically, however, this research strategy not only limits understanding cognition’s larger role in intelligence and behavior, it runs the risk of distorting it. A good case can be made that isolating cognition as a module led to the view that cognition is essentially an engine that operates on amodal symbols. If cognition had been examined within the larger context of the Situated Action Cycle from the start, it might well have taken a much different, perhaps more grounded, form from the onset of the Cognitive Revolution.

A similar case can be made that even much work on grounded cognition tends to ignore the Situated Action Cycle, addressing cognition within a relatively narrow perspective (including much of my work). In particular, research on multimodal simulation tends to focus on how simple perceptual stimuli, such as pictures and words, activate conceptual and semantic representations. Although these representations are often grounded in sensory-motor systems (thereby engaging part of the Situated Action Cycle), they are often not framed within the larger context of affect, action, and outcomes (thereby ignoring the majority of the cycle).

Thus, one future challenge for grounded cognition is to examine cognition thoroughly within the full context of the Situated Action Cycle. Besides establishing how cognition creates self-relevance as agents engage with their environments, future research could increasingly establish how cognition permeates emotion, how cognition conceptualizes action and guides it, and how cognition interprets reward and feedback. Clearly much work already research addresses these issues, including research on emotion (e.g., Barrett, 2017; Barrett & Russell, 2015; Gendron & Barrett, 2009; Lebois et al., 2018; Wilson-Mendenhall & Barsalou, 2016), research on action (e.g., Barsalou, 2016a; Hommel, Müsseler, Aschersleben, & Prinz, 2001; Rosenbaum, Carlson, & Gilmore, 2001), and research on reward (e.g., Bickel, MacKillop, Madden, Odum, & Yi, 2015; Moreira & Barbosa, 2019). Nevertheless, much more remains to be learned about how cognition becomes integrated with all phases of the cycle. Such research would further establish how cognition becomes grounded across all relevant domains (not just in embodiment), and how all phases of the Situated Action Cycle work together to produce intelligence and behavior.

Health behaviors offer one natural set of phenomena for examining the Situated Action Cycle in this manner (e.g., eating, drinking, habits, stress). Consider the cycle’s role in eating (e.g., Chen, Papies, & Barsalou, 2016; Papies, 2017; Papies & Barsalou, 2015). The environment plays major roles in stimulating and controlling eating, although self-relevance related to pleasure, health consequences, identity, and social norms plays major roles as well. Emotion and motivation are also central, together with diverse actions ranging from simple acts of consumption to connecting socially with others. Consequential outcomes follow from these actions, such as obesity, longevity, and social networks. Understanding the cognition of eating requires grounding it in all phases of the Situated Action Cycle. It is difficult to think of any activity more basic to biological life than consumption. Understanding the cognition associated with eating would constitute a major contribution to understanding intelligence and behavior and might well serve as a model for addressing other basic forms of cognition embedded in situated action.

## 3. Moving beyond viewing embodiment as only action

Embodiment is often equated with action, including motor actions, eye movements, and facial expressions. Action is undoubtedly central to most human activity, as much literature cited in earlier sections for Grounded Cognition and the Situated Action Cycle demonstrates. Action is also undoubtedly a central form of embodiment.

Many other bodily systems, however, are also central to cognition, affect, and behavior, including the autonomic system, the endocrine system, the immune system, the cardiovascular system, the respiratory system, the digestive system, and the integumentary system. Additionally, action wouldn’t be possible without the skeletomotor system. Although many of these systems may seem irrelevant to cognition, affect, and behavior, they often contribute to them significantly and in turn are affected by them. Rather than operating in isolation, these systems often couple with other systems to implement complementary functions that support goal pursuit and survival. Reviews of these systems and their interactions can be found in the *Handbook of Psychophysiology* (Cacioppo, Tassinary, & Bernston, 2016), documenting their many connections to cognition, affect, and behavior. Consider some examples:

- The autonomic nervous system not only helps regulate bodily functions, such as heart rate, respiration, and digestion, it also contributes to cognition and social interaction, including attention (pupil dilation), emotion, flight/fight responses, and sexuality (e.g., Critchley, Eccles, & Garfinkel, 2013; Larsen & Waters, 2018; Levenson, 2014; Öhman, Hamm, & Hugdahl, 2000).
- The neuroendocrine system produces hormones that not only regulate metabolism, immune activity, and tissue function, but that also contribute to cognition, behavior, and social interaction, including cortisol in emotion and stress (e.g., Epel et al., 2018; McEwen, 2013, 2018), estrogen and testosterone in sexual behavior and cognitive processes (e.g., Hutchinson, 1995; Janowsky, 2006; Newman, Sellers, & Josephs, 2005), and oxytocin in social bonding (e.g., Bosch & Young, 2018; Gangestad & Grebe, 2017).
- The immune system not only combats bacterial and viral infections, it also plays central roles in cognition and social interaction, contributing to the processing of threat, stress, trust, social connection, loneliness, and well-being (e.g., Fredrickson et al., 2013; Inagaki, Muscatell, Irwin, Cole, & Eisenberger, 2012; Kiecolt-Glaser, Gouin, & Hantsoo, 2010; Mehl, Raison, Pace, Arevalo, & Cole, 2017; Miller, Chen, & Parker, 2011; Slavich & Cole, 2013). Increasingly, such research demonstrates that the immune system plays major roles in cognition and affect.
- The cardiovascular system not only distributes blood throughout the body, it also contributes to the cognitive functions of attention, executive processing, visuo-spatial orientation, processing speed, and workload capacity, as well as to the social functions of emotion, stress, and well-being (e.g., Chapman et al., 2013; Critchley & Garfinkel, 2018; Duschek, Muckenthaler, Werner, & Reyes del Paso, 2009; Leritz, McGlinchey, Kellison, Rudolph, & Milberg, 2011; Magnusdottir, Johannsdottir, Bean, Olafsson, & Gudnason, 2017). Heart rate variability has become an especially important cardiovascular measure, given its positive associations with many of the functions just noted (e.g., Ernst, 2017; Kim, Cheon, Bai, Lee, & Koo, 2018; Mather & Thayer, 2018; Shaffer & Ginsberg, 2017). The respiratory system contributes to many of these functions as well (e.g., Varga & Heck, 2017; Zelano et al., 2016), with Varga and Heck explicitly addressing implications for embodied cognition.
- Although it might seem like the digestive system would be largely irrelevant to cognition, affect, and behavior, it is becoming increasingly clear that the gut biome contributes significantly to all of them (Mayer, 2016), including effects on emotion, memory, and choice (e.g., Bagga et al., 2018; Galland, 2014; Manderino et al., 2017; Perry & Grace, 2015).
- Besides constituting the sensory system for haptic information (an important perceptual function), the skin in the integumentary system carries information about cognition and emotion via electrodermal activity, as frequently assessed with measures such as the Skin Conductance Response, SCR (e.g., Boucsein, 2012; Prokasy, 2012).

When cognition is viewed from the perspective of the Situated Action Cycle, it becomes clear why all these basic bodily systems are important. If cognition’s relations to affect, action, and outcomes are examined fully, it becomes clear that all these basic bodily systems play fundamental roles. The cycle provides a natural means of motivating the importance of these systems for cognition, affect, and behavior, and for explaining how their coupled interactions support goal pursuit and survival. If future research is to adequately understand and explain embodiment, all these systems must be included in theory and empirical assessment. Focusing on bodily action is just the tip of the iceberg.

## 4. Deepening our understanding of multimodal simulation

Multimodal simulation is perhaps one of the most researched and controversial topics associated with grounded cognition. Considerable evidence exists that the sensory-motor modalities become active as people process conceptual and semantic information (for examples of reviews and relevant collections, see (Barsalou, 1999, 2008a, 2016b; Coello & Fischer, 2016a, 2016b; De Vega et al., 2008; Kemmerer, 2015, 2019; Kiefer & Barsalou, 2013; Kiefer & Pulvermüller, 2012; Martin, 2007, 2016; Meteyard, Cuadrado, Bahrami, & Vigliocco, 2012; Pecher & Zwaan, 2005; Pulvermüller, 1999, 2005, 2013). Importantly, however, it’s not clear what functions these activations play, and whether other forms of representation are active as well (for examples of articles that address these issues, see Barsalou, 2016b; Dove, 2009; Machery, 2007; Mahon, 2015; Mahon & Caramazza, 2008; also see the special issue of *Psychonomic Bulletin & Review*, 2016, pp. 941–1143).

Understanding the general set of issues raised by research on multimodal simulation is not only important for understanding conceptual processing and the semantics of language, it is also central for understanding cognition more generally. As suggested in a moment, multimodal simulation appears to permeate all phases of the Situated Action Cycle, not just the self-relevance phase. To the extent that simulation and other forms of knowledge representation permeate grounded cognition—supporting its myriad functions—the field will not move forward significantly until knowledge representation becomes well-understood.

One important challenge for research on multimodal simulation is to move beyond simply demonstrating that sensory-motor processes and brain areas become active during conceptual and semantic tasks. Instead, more mechanistic accounts of the specific processes and networks that underlie simulation must be developed and assessed. Already significant progress has taken place, as researchers target specific mechanisms in empirical research (e.g., Edmiston & Lupyan, 2017; Liu, Dolan, Kurth-Nelson, & Behrens, 2019; Ostarek & Huettig, 2017a, 2017b; Ostarek, Ishag, Joosen, & Huettig, 2018; Ostarek, Joosen, Ishag, de Nijs, & Huettig, 2019) and in computational models (e.g., Adams, Wennekers, Cangelosi, Garagnani, & Pulvermuller, 2014; Blouw, Solodkin, Thagard, & Eliasmith, 2015; Caligiore, Borghi, Parisi, & Baldassarre, 2010; Eliasmith, 2013; Garagnani & Pulvermüller, 2016; Pulvermüller, Garagnani, & Wennekers, 2014). Ostarek and Huettig (2019) present a variety of important issues associated with making progress in this research area.

As the mechanisms that produce multimodal simulations become increasingly established, it will be essential to continue assessing the functional roles that simulations play. Are they causal mechanisms that influence cognition, affect, and behavior? Or are they simply epiphenomena? Do they function representationally, explicitly conveying information about what they represent, informing later processes that utilize this information? Or do they operate more implicitly, just streamlining sensory-motor processing in various ways and only influencing higher-order cognitive processes associatively?

Increasing evidence demonstrates that multimodal simulations play causal roles in cognition, coming from both lesion research (e.g., Cotelli, Manenti, Brambilla, & Borroni, 2018; Kemmerer, Rudrauf, Manzel, & Tranel, 2012; Riccardi, Yourganov, Rorden, Fridriksson, & Desai, 2019a, 2019b; Urgesi, Candidi, & Avenanti, 2014) and from brain stimulation research (e.g., Pulvermüller, Hauk, Nikulin, & Ilmoniemi, 2005; Repetto, Colombo, Cipresso, & Riva, 2013; Vukovic, Feurra, Shpektor, Myachykov, & Shtyrov, 2017; Vukovic & Shtyrov, 2019). Kemmerer (2019) provides a review of relevant literature.

Another key issue will be assessing whether amodal symbols accompany multimodal simulations, perhaps playing central roles (e.g., Dove, 2009; Machery, 2007; Mahon, 2015; Mahon & Caramazza, 2008). A related issue is whether distributed linguistic representations play central roles as well (e.g., Andrews, Frank, & Vigliocco, 2014; Andrews, Vigliocco, & Vinson, 2009; Barsalou, Santos, Simmons, & Wilson, 2008; Connell & Lynott, 2013; Glaser, 1992; Louwerse, 2008; Louwerse & Connell, 2011; Paivio, 1986; Vigliocco, Meteyard, Andrews, & Kousta, 2009). In some cases, use of linguistic representations may explain evidence that has been interpreted as supporting amodal theories (e.g., Barsalou, 2016b; Barsalou et al., 2008; Glaser, 1992; Louwerse & Connell, 2011; Solomon & Barsalou, 2004). Many researchers believe that knowledge representation is a complex multifaceted process, using multiple forms of representation. To the extent that this view is correct, it will not only be important to establish accounts of operative representations, but even more important to establish how they work together to implement intelligence and situated action.

Still another central topic concerns the roles of the brain’s association areas during conceptual and semantic processing (Barsalou, 2016b). Hub and spoke theories propose that association areas in the anterior temporal lobes function as hubs that integrate semantic information in the modalities. Considerable evidence has accumulated for this view (Chen, Lambon Ralph, & Rogers, 2017; Lambon Ralph, Jefferies, Patterson, & Rogers, 2017), with accompanying computational models (e.g., Hoffman, McClelland, & Lambon Ralph, 2018; Rogers et al., 2004). Other accounts emphasize additional association areas in the angular gyrus, middle temporal gyrus, and cortical midline (e.g., Binder, Desai, Graves, & Conant, 2009; Binder et al., 1999; Binder, 2016; Martin, 2016). Increasing evidence demonstrates that activations in association areas carry semantic—and even sensory-motor—information (e.g., Fernandino et al., 2016, 2015).

One possibility is that activations in association areas function as explicit representations. If so, then a key question is whether these representations can function on their own without accompanying sensory-motor simulations, or whether they only act in concert with simulations. Can information be read off representations in association areas and used for diverse purposes without sensory-motor representations being active? An alternative possibility is that activations in association areas primarily function as triggers for controlling sensory-motor simulations without actually functioning as explicit representations (Damasio, 1989; also see Simmons & Barsalou, 2003).

In Kuhnke, Kiefer, and Hartwigsen (2020), for example, both modality-specific and association areas became active as people shifted attention between auditory and motor features during conceptual processing. It’s not clear, however, where information about the properties used for the task was sourced. Was it obtained from the relevant modality-specific areas, related association areas, or both? If only the association areas had been active, could the task have been performed—could the information needed have been read out of these activations alone? Considerable challenges and opportunities exist for establishing the content and function of activations in association areas during conceptual and semantic processing. More generally, we will not understand the fundamental nature of knowledge representation in cognition without understanding the roles of these areas.

Finally, considerable opportunity exists for understanding the diverse roles of conceptual and semantic processing throughout the Situated Action Cycle (Figures 2 and 3). Most typically, research on conceptual and semantic processing examines responses to words, texts, and pictures. Although much has been learned from this work, it fails to capture the diverse roles of multimodal simulation across other phases of the cycle. For example, researchers increasingly propose that multimodal simulation plays central roles in emotion (e.g., Barrett, 2017; Lebois et al., 2018; Wilson-Mendenhall & Barsalou, 2016; Wilson-Mendenhall et al., 2011), action (e.g., Barsalou, 2016a; Grush, 2004; Wolpert & Flanagan, 2001), and outcomes (e.g., Papies & Barsalou, 2015; Papies, Best, Gelibter, & Barsalou, 2017). Rich opportunities for obtaining evidence of multimodal simulation may exist in these phases of the Situated Action Cycle, with the roles of simulation perhaps varying from its roles in conceptual and semantic processing.

## 5. Grounding symbolic processes

Symbolic processes such as type-token predication, concept composition, recursion, and propositions are often associated with modular cognition and the amodal symbols it typically champions (Fodor, 1975; Fodor & Pylyshyn, 1988; Pylyshyn, 1973, 1984). Conversely, symbolic processes are typically *not* associated with grounded cognition, and might indeed be viewed as antithetical to its general approach. Such thinking sometimes includes the assumption that symbolic processes can only result from the processing of amodal symbols. For some time, however, it has been well-known that symbolic processes can emerge from other kinds of mechanisms, including images (Price, 1953) and neural nets (Smolensky, 1990).

In developing Perceptual Symbol Systems—a theory whose primary focus was explaining symbolic processing from a grounded perspective—Barsalou (1999) agreed with classic amodal theorists that symbolic processes are central to cognition in general (Fodor & Pylyshyn, 1988), and to the processing of images in particular (Pylyshyn, 1973). Barsalou (1999) further reviewed how symbolic processes are not unique to amodal symbols but can operate in a wide variety of representational contexts. Finally, the central ideas of Perceptual Symbol Systems revolved around simulation-based accounts of symbolic processing, providing alternative accounts of type-token predication, concept composition, recursion, and propositions. Barsalou (2003a, 2005, 2017a) developed this approach further, and Barsalou (2008b) reviewed empirical evidence for it.

For any approach to explain symbolic processing, it must have a means of implementing concepts, where a concept is a mechanism that aggregates information accumulated from experiences with a category’s members (also known as a *type*). For example, the concept of pizza aggregates information accumulated from encounters with pizza instances (tokens) to represent this type of thing in the world. Although many theories assume that a concept is a fixed abstraction (e.g., a definition or prototype of pizza), an alternative approach is that a concept is a competence or disposition for generating infinite conceptualizations of a category (e.g., infinite conceptualizations of pizza; Barsalou, 1987, 1989, 1993, 1999; Casasanto & Lupyan, 2015; Connell & Lynott, 2014; Lebois, Wilson-Mendenhall, & Barsalou, 2015; Yee & Thompson-Schill, 2016).

To implement the latter approach from the perspective of grounded cognition, Perceptual Symbol Systems developed a distinction between *simulators* and *simulations*. Whereas the entire body of accumulated knowledge for a category constitutes a *simulator*, using the simulator to construct a conceptualization on a specific occasion constitutes a *simulation*. Consider how an individual might establish a simulator for the category of pizza. As the individual consumes pizza on a specific occasion, brain areas that process the pizza’s features become active to represent them in the relevant modalities (Chen et al., 2016), with association areas integrating these modality-specific representations (e.g., Barsalou, 2016b; Barsalou et al., 2018; Binder, 2016; Fernandino et al., 2016; Simmons & Barsalou, 2003). For example, brain areas that process how pizzas look, taste, smell, and feel might become active, as well as areas that process actions, emotions, and rewards associated with consuming them. In other words, all the areas required to implement the Situated Action Cycle with pizza become active, together with relevant association areas. On each occasion when pizza is consumed, a distributed associative pattern becomes established across these neural systems. Across many episodes of consuming pizza, an increasingly entrenched associative network emerges throughout the brain, accumulating the aggregate results of superimposing pizza information on relevant neural systems time after time. The sloppy, difficult-to-localize brain network that evolves into a pizza simulator essentially implements a pizza concept, given that it contains accumulated information about pizza that can be used to represent pizza in its absence.

Once simulators exist for categories, they enable symbolic processes (Barsalou, 1999, 2003a, 2005, 2017a). For example, binding the pizza simulator to a perceived object during perception establishes a type-token predication of the object, essentially expressing the proposition that it is token of the type pizza, further enabling rich conceptual inferences about it. To the extent that the object actually turns out to be a pizza, the proposition is true. Conversely, if the object turns out to be flatbread, the proposition is false.

Similarly, one form of concept composition results from binding multiple simulators to multiple perceived entities in the world and then relating them together with a relational simulator. The proposition that an airplane is above a cloud, for example, can be represented by first binding simulators for airplane and cloud to perceived objects, and then binding these objects to spatial regions in the relational simulator for above (Barsalou, 1999, Figure 5). Extending this approach to the Situated Action Cycle, Barsalou et al. (2018) proposed that integrating components of situations during situated action constitutes one fundamental form of concept composition (with another fundamental form arising in language that describes situated action).

For whatever reason, the simulator-based accounts of symbolic processing in Perceptual Symbol Systems haven’t received much attention—positive or negative. Instead, research following from the Perceptual Symbol Systems framework has focused on claims about multimodal simulation in conceptual and semantic processing. If one assumes, however, that cognition does not contain an amodal symbolic engine but does revolve around symbolic processing, then it might be useful to explore the possibility that symbolic processing is implemented with simulation mechanisms. No doubt the preliminary theoretical accounts of symbolic processing in Perceptual Symbol Systems must be improved upon and/or developed substantially, along with a stronger empirical case for them (cf. Barsalou, 2008b; Solomon & Barsalou, 2001; Wu & Barsalou, 2009). Nevertheless, this may be a potentially important direction for future research.

A likely possibility is that alternative approaches to grounding symbolic processing will develop that differ significantly from the grounded approach described here. Still another possibility is that non-grounded approaches may offer effective accounts, such as those found in Werning, Hinzen, and Machery (2012), Winter and Hampton (2017), Pylkkänen (2019), and the 2019 special issue on meaning composition in *Philosophical Transitions of the Royal Society B* (Martin & Baggio, 2020).

## 6. Grounding abstract concepts

Another common misconception about grounded cognition is that it can explain concrete concepts but not abstract concepts. Although multimodal simulations might play a role in the representation and processing of concrete concepts (e.g., hammer), how could they possibly explain concepts that lack sensory-motor content (e.g., truth)? Because of this concern, a long-standing tradition states that abstract concepts are represented via language (Binder, Westbury, McKiernan, Possing, & Medler, 2005; Paivio, 1986; but see Wilson-Mendenhall, Simmons, Martin, & Barsalou, 2013). Actually, this proposal about the importance of language explains little about the semantics of abstract concepts (although language certainly does appear central for processing them; Barsalou et al., 2018; Borghi et al., 2017). Exactly how linguistic forms represent the semantics of abstract concepts has never been made clear, and many seriously doubt that linguistic forms are sufficient for doing so—conceptual processes of some sort are necessary.

All theories of cognition and conceptual processing struggle with abstract concepts. Not only are they a challenge for the grounded perspective, they are a challenge for all perspectives, including the amodal approach. Find *any* theory, including an amodal one, that offers an illuminating and satisfying account of abstract concepts! Simply proposing that amodal symbols must represent abstract concepts because they can’t be grounded in sensory-motor systems is uninformative about their specific semantics. Not much is learned from this negative definition (Barsalou et al., 2018). A positive definition of the semantics that abstract concepts contain—not the semantics they don’t contain—is a necessary first step to explaining them.

The grounded approach has actually offered many concrete proposals about the semantics of abstract concepts. Cognitive linguistics offered a metaphor theory of abstract concepts, grounding them in bodily and other experiential schemata (e.g., Lakoff, 1987; Lakoff & Johnson, Mark, 1980). My colleagues and I suggested that abstract concepts originate in both introspective experience and in the integration of situational components (Barsalou, 1999; Barsalou et al., 2018; Barsalou & Wiemer-Hastings, 2005; Wilson-Mendenhall et al., 2013). Barsalou et al. (2018) proposed that we should drop the distinction between concrete and abstract concepts. On the one hand, it defines abstract concepts as not concrete (a negative definition), which offers virtually no insight into their semantics. On the other hand, abstract concepts contain much concrete information, and concrete concepts contain much abstract information. The distinction between concrete and abstract concepts may actually confuse and distort our understanding of these concepts more than enlighten us.

Barsalou et al. (2018) suggest that a more productive approach is to address the roles of specific concepts within the context of processing situations, from the perspective of the Situated Action Cycle (Figures 2 and 3). Whereas traditional concrete concepts often represent elements of the external environment, action, and outcome phases, traditional abstract concepts often represent elements of the internal self-relevance and affect phases. Additionally, other abstract concepts integrate patterns of concepts across phases of the Situated Action Cycle. Rather than describing concepts operating in different phases as concrete or abstract, it may be more useful to specify their specific roles in supporting the cycle, and how they pattern with other concepts to do so. Much recent work is consistent with this account, finding that abstract concepts often include features for internal experience and event integration, such as features for thought, time, interoception, quantity, emotion, social interaction, and morality (e.g., Binder et al., 2016; Connell, Lynott, & Banks, 2018; Crutch, Troche, Reilly, & Ridgway, 2013; Harpaintner, Trumpp, & Kiefer, 2018; Troche, Crutch, & Reilly, 2014; Vargas & Just, 2020; Villani, Lugli, Liuzza, & Borghi, 2019; Wang, Wang, & Bi, 2019). Barsalou (2017b, Section 3.1.2) raises issues associated with the methods used to identify the specific features in this research.

To the extent that this approach has merit, then another challenge for grounded cognition is establishing how concepts of all types pattern together to support the Situated Action Cycle. Whereas some concepts represent relevant elements of the cycle in the external world, others represent relevant elements of the cycle in the internal world. Still other concepts integrate concepts spanning both the external and internal worlds as the Situated Action Cycle unfolds. Understanding how different kinds of concepts work together to process situations and implement situated action may perhaps offer leverage for finally making significant progress on establishing the semantics of abstract concepts. Assessing their semantics in isolation as responses to words is unlikely to teach us much, whereas understanding their roles in the Situated Action Cycle may allow us to crack the code.

## 7. Individual differences in grounded cognition

As described earlier, each run of the Situated Action Cycle superimposes a trace of its activity on the brain and body, leaving behind a distributed associative pattern of the neural and bodily systems assembled to implement the cycle on that occasion (Figure 2). As a result of this repetitive conditioning, entrenched patterns develop that implement habitual ways of thinking, feeling, and acting in frequently-experienced situations (Barsalou, 2003b, 2016c, 2016d; Barsalou et al., 2003; Lebois et al., 2018).

This account naturally predicts substantial individual differences in grounded cognition, including individual differences associated with culture (Kemmerer, 2019). To the extent that individuals implement the Situated Action Cycle differently—as a function of their genes, parenting, resources, physical environment, social environment, culture, and so forth—they establish widely different habits from using the cycle across situations, including habits for eating, drinking, exercising, socializing, working, using technology, sustaining the environment, regulating emotion, mentalizing, and many others (Barsalou, 2019; Dutriaux et al., 2019).

To see the potential for individual differences in these common activities, consider each phase of the Situated Action Cycle. In the environment phase, individuals experience different physical, social, and cultural environments. In the self-relevance phase, individuals experience different goals, values, norms, and identities. In the affective phase, individuals experience different emotions and motivations, along with different strategies for regulating them. In the action phase, individuals perform different actions, especially during socio-cultural activities. In the outcome phase, individuals experience different patterns of immediate and long-term reward, punishment, and so forth. Within the extensive space of what can be implemented during phases of the Situated Action Cycle, tremendous possibilities arise for individual differences, along with how the brain and body become conditioned as a consequence.

From a population perspective, individual differences result from each individual establishing a unique population of situational memories in their brain and body (what we have referred to as *populations of situated conceptualizations*). As individuals diverge in the use of the Situated Action Cycle during routine habits, different populations of situational memories accumulate. For example, as individuals engage the cycle differently during eating, they establish different populations of eating memories that condition future eating behavior (Barsalou, 2016c, 2016d; Chen et al., 2016; Papies & Barsalou, 2015; Papies et al., 2017). To the extent that the Situated Action Cycle grounds cognition (Figures 1, 2 and 3), it follows that large individual differences in grounded cognition should be the rule, not the exception. Consistent with the existence of such a rule, large individual differences occur in the situated activity associated with health behaviors (e.g., Barsalou, 2019; Dutriaux et al., 2019; Taylor Browne Luka, Dutriaux, Hendry, Stevenson, & Barsalou, 2019; Werner et al., 2019). Large individual differences also occur in neural activity as people perceive complex everyday events associated with situated action (e.g., Huth, de Heer, Griffiths, Theunissen, & Gallant, 2016; Huth, Nishimoto, Vu, & Gallant, 2012; Schmälzle, Imhof, Grall, Flaisch, & Schupp, 2017).

If this reasoning is correct, then incorporating individual and cultural differences centrally into grounded cognition offers another challenging direction for future research. Rather than simply addressing common grounding mechanisms across individuals and cultures, differences between them must be addressed as well. On the one hand, individual and cultural differences offer considerable opportunity for understanding the nature of grounding mechanisms—and indeed may be necessary for doing so. On the other, addressing these differences offers the grounded approach a platform for addressing socially relevant issues, including individual and cultural differences in physical health, mental health, technology use, sustainable behavior, work, education, social interaction, and so forth.

## 8. Replication, generalization, and quantum mechanisms

The failure of research findings to replicate is of much current interest (Bollen et al., 2015). General agreement exists that no single factor produces replication failure. Instead multiple factors are responsible, including poor methodological practices, weak power, problematic statistical procedures, and incomplete reporting policies.

In the literature on grounded cognition, high-profile findings sometimes fail to replicate, including the action compatibility effect (ACE) and the facial mimicry effect (Morey et al., 2020; Wagenmakers et al., 2016). Other grounded effects, however, have replicated, including the spatial compatibility effect (Zwaan & Pecher, 2012) and the spatial interference effect (Estes & Barsalou, 2018). Importantly, replicable effects often exhibit considerable context sensitivity, with the presence of contextual moderators being important for their presence (Estes & Barsalou, 2018; Van Bavel, Mende-Siedlecki, Brady, & Reinero, 2016).

Context sensitivity may be much more widespread than generally assumed, challenging our ability to generalize mechanisms identified in the laboratory to real-world situations (e.g., Barsalou, 2019; Brunswik, 1947, 1955; Hammond & Stewart, 2001; Miller et al., 2019). Phenomena often assumed to be robust across task conditions may actually be context sensitive, not generalizing across situations. Consider automaticity as exemplified by Stroop interference and Simon congruency. Perhaps surprisingly, much research finds that these and other classic automaticity effects exhibit context sensitivity (Gawronski & Cesario, 2013; Kiefer, Adams, & Zovko, 2012; Lebois et al., 2015; Meteyard & Vigliocco, 2018). If a mechanism like automaticity is context sensitive, it would not be surprising if many other classic cognitive mechanisms are context sensitive as well, such as frequency, visual search, repetition priming, syntactic priming, the availability heuristic, and so forth. The ideal of robust context-independent mechanisms that generalize across situations may not only be a myth, but an obstruction to understanding how cognition and the brain operate.

Adopting an informal quantum perspective on mechanisms offers a useful lens for examining replicability and generalizability (Barsalou, 2019; for formal approaches to quantum cognition, see Bruza, Wang, & Busemeyer, 2015; Pothos & Busemeyer, 2013; Wang, Busemeyer, Atmanspacher, & Pothos, 2013). From an informal quantum perspective, a psychological mechanism takes infinitely many forms across situations—not a single constant form. Although the mechanism’s expression varies, these expressions nevertheless exhibit a central tendency that constitutes its default form. When the quantum mechanism is reduced to a classical mechanism, its default form becomes the mechanisms’ classical constant form. From a quantum perspective, when all other factors are equal, the default form is most likely to emerge when the mechanism becomes engaged. In actual practice, though, contextual moderators typically influence the mechanism’s expression, increasing the likelihood of a contextually relevant form. As a result, different contextual conditions often drive the mechanism into contextually relevant forms that differ from the default (i.e., with the default maintaining a presence, in the Bayesian spirit of combining the mechanism’s prior with the situation’s likelihood). The quantum perspective also assumes that a mechanism can be in multiple simultaneous states to varying degrees and that observing the mechanism influences its measurement, with objective measurement being impossible.

Consider Barsalou’s (2019) example of simulators as quantum mechanisms. As described earlier, a simulator aggregates multimodal experiences with a category across situations to implement a concept. For example, experiences of consuming pizza establish a pizza simulator that functions as a concept for the category of pizza. Once a pizza simulator exists, it produces multimodal simulations of pizza in relevant situations. When a cue in the current situation activates the simulator (e.g., a perceived pizza, the word “pizza,” a relevant pizza setting), a very small subset of the simulator’s distributed network becomes active—a simulation—to create one possible representation of the category. As different subsets of the pizza simulator become active across situations, different simulations express different conceptualizations.

Across situations, an infinite number of pizza simulations are possible, such that the pizza concept is expressed in a quantum manner. Extensive research documents the extensive variability in how the ‘same concept’ is represented across situations dynamically (Barsalou, 1987, 2016b, 2017b; Casasanto & Lupyan, 2015; Connell & Lynott, 2014; Lebois et al., 2015; Meteyard & Vigliocco, 2018; Yee & Thompson-Schill, 2016). Additionally, however, a central tendency exists across possible pizza simulations that constitutes the pizza simulator’s default simulation. At the classical level, the simulator’s default constitutes the fixed pizza concept that researchers often assume and attempt to measure. At the quantum level, the simulator constitutes the pizza concept, expressing itself in infinite ways across situations. Finally, multiple simulations of pizza could be simultaneously active (or partially active) within the simulator at a given moment. and measuring the pizza simulator (or one of its simulations) influences its expression.

Barsalou (2019) discusses implications of the quantum perspective for the ability to replicate experimental findings (also see Yarkoni, 2019). In particular, replication failures may often result from the fact that quantum mechanisms are being observed that express themselves differently across occasions, even when direct replication is attempted using best practices. To the extent that a new experimental situation differs from the original one, it becomes increasingly likely that the original finding will not generalize. Furthermore, generalization failure is especially likely when mechanisms identified in the laboratory are assessed in the real world (Barsalou, 2019; Brunswik, 1947, 1955; Hammond & Stewart, 2001; Miller et al., 2019). Quantum mechanisms studied in the laboratory may often take relatively rare and exotic forms, relative to the more likely forms they normally take outside the laboratory.

Again, many other factors underlie replication failure besides context sensitivity, including poor methodological practices, weak power, problematic statistical procedures, and incomplete reporting policies. No doubt, we must replace poor research practices with best practices. Nevertheless, if we are dealing with quantum mechanisms, then this needs to be factored into best practices as well. Practice informed by naïve theory is not best practice.

Thus, another challenge for grounded cognition is to understand the quantum nature of not only grounding mechanisms, but of cognitive, affective, behavioral, and neural mechanisms in general. Learning how to measure and generalize these mechanisms experimentally constitute further challenges. Taking a population approach to the situational memories established by the Situated Action Cycle offers one approach for understanding, representing, and measuring context-sensitivity at both the group and individual levels (Barsalou, 2016c, 2016d, 2019). As the population of situational memories related to a task varies across individuals and experiments (along with the specific memories activated on specific occasions), quantum mechanisms are increasingly likely to express themselves in different ways. Assuming that these mechanisms should always express themselves identically across situations is not realistic. Embracing quantum variability and dealing with it is not only necessary for establishing successful psychological science and neuroscience, but is also likely to produce fundamental insights into cognition, affect, behavior, and the brain.

## 9. Conclusions

The Situated Action Cycle offers considerable leverage for understanding cognition, affect, behavior, and the brain—indeed it may be indispensable for doing so. Studying an element of the cycle in isolation—such as cognition—runs the risk of producing a limited and distorted account. As proposed earlier (Figures 1, 2 and 3), the Situated Action Cycle integrates the domains of grounded cognition, most likely, because each domain is necessary for fully capturing intelligence and situated action. Within this context, embodiment is more than bodily responses to faces and words, becoming instead the broad panorama of bodily systems required to implement the cycle in all its phases. Similarly, multimodal simulation is more than conceptual responses to perceived objects and words, becoming instead a process that develops through use of the Situated Action Cycle to subsequently support all its phases. Additionally, understanding symbolic processes and abstract concepts may become more tractable once we observe how they emerge from the cycle and in turn support it. Finally, individual differences can be understood as the natural outcome of the cycle establishing quantum mechanisms via aggregation of unique populations of situational memories in different individuals, with replication and generalization reflecting the match of these individual populations to the situations where they’re assessed.

Approaching cognition through the lens of the Situated Action Cycle also encourages the understanding of cognition, affect, and behavior in relatively naturalistic forms, rather than in highly idealized laboratory forms whose connection to real-world phenomena may be questionable (e.g., Barsalou, 2019; Brunswik, 1947, 1955; Hammond & Stewart, 2001; Miller et al., 2019). Certainly, it’s essential to use idealized laboratory paradigms for isolating and establishing causal mechanisms. Importantly, however, these paradigms are well-motivated when they shed light on important real-world phenomena, not when they’re only convenient, allow experimental control, and support sophisticated modeling. Without a doubt, convenience, control, and modeling are highly desirable features of a laboratory paradigm and of science in general. If, however, a paradigm doesn’t generalize to real-world phenomena and offer insight into them, the time and resources required for implementing it may ultimately have little justification or impact.

Although grounding cognition in the domains that underlie the Situated Action Cycle is often viewed as relatively unorthodox and controversial, I suspect that integrating grounding mechanisms into classic research will ultimately be relatively painless (Barsalou, 2010, 2016b). From this perspective, grounded cognition will not replace classic cognition but will become integrated with it. Perhaps one significant change will be the increased use of multimodal simulation as a representational process. Perhaps another will be increased embedding of cognition in the Situated Action Cycle. As this integration develops, I suspect that many classic cognitive phenomena and mechanisms will remain, continuing to play the important roles they’ve always played. Other important new developments will no doubt come to play central roles as well.

Perhaps the most important question will be how successful we are at making progress on the kinds of issues raised here. How successfully have we grounded cognition in the modalities, the body, the physical environment, and the social environment? How successfully have we captured cognition’s roles in the situated action cycle? How well have we captured the multifaceted nature of knowledge representation, including the different types of representation involved and their integrated operation? How much have we advanced our understandings of symbolic processes and abstract concepts? How well have we explained group-level mechanisms (perhaps in a quantum manner) and the substantial individual differences in their implementation on specific occasions? How much leverage does the progress we’ve made on all these fronts help us understand everyday human behavior and put us in a position to change it constructively. To the extent that we move forward on these fronts, it will not matter whether we take a grounded approach, a classic approach, or some other approach. Perhaps, however, grounding will become a foundational component of approaches that continue to emerge from psychology, cognitive science, and neuroscience over the coming decades.

## Author Information

I am grateful to Markus Ostarek and Falk Huettig for the opportunity to participate in this special issue, and to David Kemmerer and Lotte Meteyard for careful constructive reviews that improved this article significantly.
